# Establishment of reference intervals for Platelet Function Analyzer -100 closure time in Algerian adults

**DOI:** 10.1371/journal.pone.0249402

**Published:** 2021-04-12

**Authors:** Malika Belkacemi, Yassine Merad, Mohamed Amine Merbouh

**Affiliations:** 1 Blood Transfusion Department « Hassani Abdelkader » Hospital, University Center, Sidi-Bel Abbès, Algeria; 2 Faculty of Medicine, Djellali Liabes University, Sidi-Bel Abbès, Algeria; 3 Central Laboratory, « Hassani Abdelkader » Hospital, University Center, Sidi-Bel Abbès, Algeria; 4 Department of Epidemiology and Preventive Disease « Hassani Abdelkader » Hospital, University Center, Sidi-Bel Abbès, Algeria; University of Witwatersrand/NHLS, SOUTH AFRICA

## Abstract

**Introduction:**

The Platelet Function Analyzer-100 (PFA-100) is a point of care instrument that simulates plug formation under high shear flow. The PFA-100 measures the time required to occlude the aperture in a biochemically active cartridge and is expressed in a term of closure time (CT). In Algeria, the reference values used in clinical laboratories are of Western origin. However, ethnic, genetic, dietary environmental, and diet differences between populations may affect reference intervals. We established the reference intervals of PFA-100 closure times in healthy Algerian adults according to the International Federation of Clinical Chemistry method, and we compared them with those of Western and Asian countries.

**Material and methods:**

We enrolled 303 healthy blood donors in the study. 218 subjects met inclusion criteria. We analyzed the blood sample on the PFA-100 for CT with both the collagen epinephrine and collagen ADP cartridges.

**Results:**

The reference intervals of PFA-100 collagen epinephrine CT and PFA-100 collagen ADP CT were 91–207 seconds and 71–144 seconds, respectively. Compared to Western and Asian populations, there were significant differences. The upper limits of CTs were higher for Algerians in this study. Our findings show that many healthy Algerians would be incorrectly identified as having a primary hemostasis abnormality according to the reference intervals of the manufacturer and scientific literature.

**Conclusion:**

This report provides the first reference intervals for PFA-100 CTs in healthy Algerian adults. These results improve the accuracy of diagnosis and patient care in Algeria.

## Introduction

The PFA-100 is a point of care instrument for hemostasis testing. Citrated whole blood passes at high shear rates through disposable cartridges containing an aperture in a membrane coated with Collagen and Epinephrine (CEPI) or Collagen and ADP (CADP) [1]. These agonists, with the high shear stress, induce a plug formation in and around the aperture. The time required to occlude the aperture and to stop blood flow is called the closure time (CT). The PFA-100 is an automated alternative to the bleeding time (BT) test, which is no longer recommended [2]. The PFA-100 allows rapid and global evaluations of primary hemostasis. Recently, we equipped our laboratory with this instrument. However, there are few studies on the reference intervals (RIs) for PFA-100 CTs. In addition, most of the studies do not meet international recommendations such as defined in the International Federation of Clinical Chemistry (IFCC) and Clinical and Laboratory Standards Institute (CLSI) guidelines. All the previous studies were conducted in the Western or Asian populations. In Algeria, clinical laboratories use the reference intervals (RIs) of PFA-100 provided by manufacturers which are of Western origin. We know that ethnic, genetic, and dietary differences between populations may affect RIs. So far, we have not come across any literature on Algerian reference values for PFA-100 CT. To respond to this lack, we decided to determine the RIs of the PFA-100 CT in healthy Algerian adults. We then compared the findings with those available in the Western and Asian healthy populations.

## Material and methods

### Selection of healthy subjects and study design

This prospective study was conducted at the blood transfusion department of the Sidi Bel Abbes City. The choice of donors is founded on the possibility that the population is supposedly in good health and that it receives a medical selection. The doctor who allows or prohibits the donation completes an information sheet. It includes personal information such as sex and age, a history of allergy, viral, bacterial or parasitic infectious disease, stays abroad, transfusion, surgery, medication or vaccination. Consent was obtained for all participants. We included in our study any Algerian adult male and female able to donate after the medical consultation. The criteria for inclusion and non-inclusion are those of blood donors. Exclusion criteria are positive HIV serology, positive HCV serology, positive HBS Ag serology, positive syphilis serology, taking aspirin or a nonsteroidal anti-inflammatory drug (NSAID), antibiotic or other drugs with a potential to influence platelet function within ten days before collection, platelet count below 150G / L or hematocrit less than 0.35 or a von Willebrand factor Antigen (vWF Ag) < 50%. The study was approved by the local ethics committee of the Hospital University Center of Sidi Bel Abbes. A minimum of 120 subjects is recommended by CLSI to establish Reference interval values [3]. 303 healthy adult (≥18 years old) were recruited. Among them, 85 were excluded: 56 for the use of aspirin or an NSAID, 14 due to hematocrit less than 0.35 and 15 because of a platelet count below 150G / L. Thus, a total of 218 subjects were included in our analysis.

### Specimen collection and processing

Blood samples were drawn from each subject at the beginning of the donation. Whole blood was collected into EDTA collection tubes for complete blood counts (CBC) determination. Blood for PFA-100 testing and routine hemostatic testing was drawn into 0.129 mol/l (3.8%) sodium citrate collection tubes. Samples for PFA-100 testing were stored at room temperature before testing. Hemolyzed or clotted samples were not used. All the samples were obtained in the morning (between 8:30 and 11:00 am). Platelet poor plasma (PPP) was obtained by centrifugation at 3500 x g for 10 min. Aliquots of 0,5 ml of PPP were snap-frozen and stored at -80C. When required for further analysis vWF: Ag samples were thawed at 37°C for 10 min.

### Assay

#### PFA-100 CT

CEPI CT and CADP CT were determined between 30 min and 1 h after blood collection, using one single lot each of CEPI and CADP cartridges by PFA-100 TM instrument from Siemens Health Diagnostics (Marburg, Germany).

#### CBC

The CBC was performed on the Sysmex XT-2000i Automated Hematology Analyzer From Sysmex Corporation, Kobe, Japan.

#### Routine hemostatic testing

Prothrombin time (PT), activated Partial Thromboplastin time (aPTT) and Fibrinogen level (Fg) were performed on the automated system STA Compact Stago from Diagnostica Stago, Asnieres, France.

#### Von Willebrand factor Antigen (vWF Ag)

vWFAg was determined by immunological method (Liatest Stago) on the automated system STA Compact Stago from Diagnostica Stago, Asnieres, France.

### Data analysis

The distribution of each variable was first inspected visually to check for normality and possible outliers. The latter were tested by Dixon’s criterion. The D’Agostino-Pearson test for normality was used to analyze the values. As not all values were normally distributed, data were presented as either median and range or as mean ± standard deviation (SD). Paired and unpaired data were compared using the Wilcoxon test and the U Whitney test, respectively. The t-test was used to compare the mean between the groups. Differences in frequencies were checked by the Chi-square test.

RIs was determined by the nonparametric method according to the IFCC and CLSI recommendations. It is based on the 2.5th and 97.5th percentile rankings and the calculation of their 90% confidence interval (CI) [4].

The RIs obtained in this study was compared to that provided by the manufacturer and other foreign studies by checking the number of our subjects who fell outside the predefined upper limit value. Differences found in proportions found were checked by Fisher’s exact test.

Statistical analyses were performed using Statistical Package for Social Science SPSS version 25 (IBM SPSS Armonk NY, USA) and, MedCalc Statistical Software version 15, 0 (MedCalc Software, Ostend, Belgium). The p value <0.05 was considered statistically significant.

## Results

### Characteristics of the reference sample

218 healthy Algerian adults (>18 years old) were enrolled in this study. Baseline data for all individuals, men and women are shown in Table 1. Females and males did not differ in age or the proportion of blood group O. Concerning smoking behaviour, as expected; the proportion of female smokers was significantly lower than that of male smokers. The distribution of subjects according to the ABO blood group was: 44% are of group O, 31% of group A, 19.8% of group B and 5.2% are of group AB. The frequency of phenotypes O, A, B, AB in the population of western Algeria is respectively 42.7%, 32.1%, 19.3%, 5.9%and therefore there was no significant difference between our sample and the population from west Algeria [5].

**Table 1:** Baseline data of 218 healthy individuals.

	All	Male	Female	p Value (M versus F)
**Number of subjects**	218	173	45	
**Age (years)**†	32 (19–61)	32 (19–61)	31 (19–61)	NS
**Smokers/Non Smokers**	55/163	54/119	1/44	< 0.0001
**Blood Group O/ Non O**	93/125	74/99	26/19	NS
**RH Positive/ RH Negative**	185/ 33	147/26	38/7	NS
**WBC (G/L)**†	6.61 (3.70–11.49)	6.61 (3.70–11.49)	6,45 (4.16–10.85)	NS
RBC (T/L)*	4.82 (0.46)	4.93 (0.41)	4.33 (0.42)	< 0.0001
Hb (g/dl)*	14.50 (1,25)	14.86 (1,04)	13,12 (1,02)	< 0.0001
**Hct** †	0.43 (0.35–0,51)	0.44 (0.35–0.51)	0.39 (0.35–0.46)	< 0.0001
**Platelet count (G/L)**†	211,50 (150–452)	210 (150–452)	218 (150-365)	NS
MPV fl*	10,41 (1,01)	10,35(0,96)	10.69 (1.19)	NS
**PT (%)**†	100 (70–100)	100 (70–100)	100 (70–100)	NS
**aPTT (sec)**†	30,60(3,99)	30,62 (4,01)	30,52 (3,96)	NS
Fg (g/l)*	3.04 (0,66)	3.01 (0.64)	3,12 (0,73)	NS
**vWF Ag (%)** †	99,76 (60–175)	99,76 (60–148)	100 (75–175)	NS

Data are expressed as median value (minimum-maximum), and Arithmetic mean (Standard Deviation)

* Student Test

† U Mann–Whitney Test

F: female, M: male

NS: Non-Significant difference

### Reference intervals

**Fig 1:**
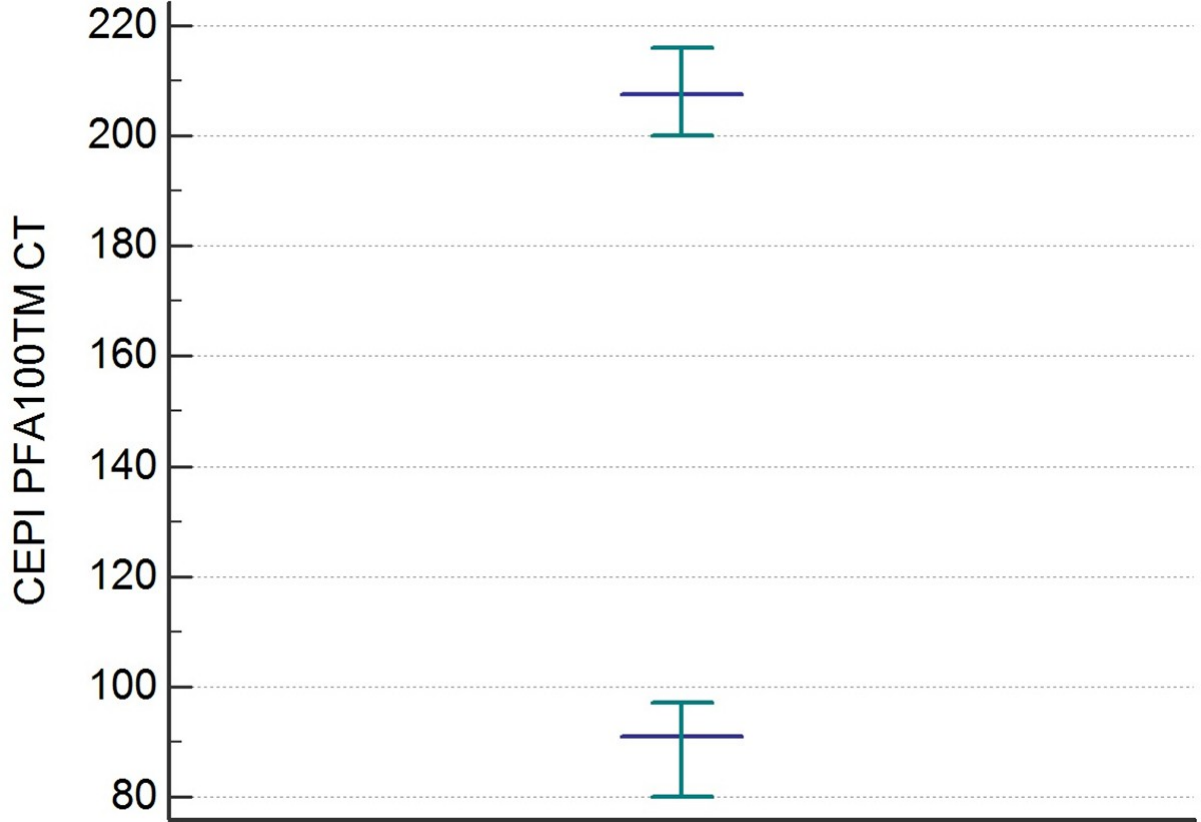
Reference intervals of CEPI PFA-100 CT determined by non-parametric percentile method (CLSI C28-A3).

**Fig 2:**
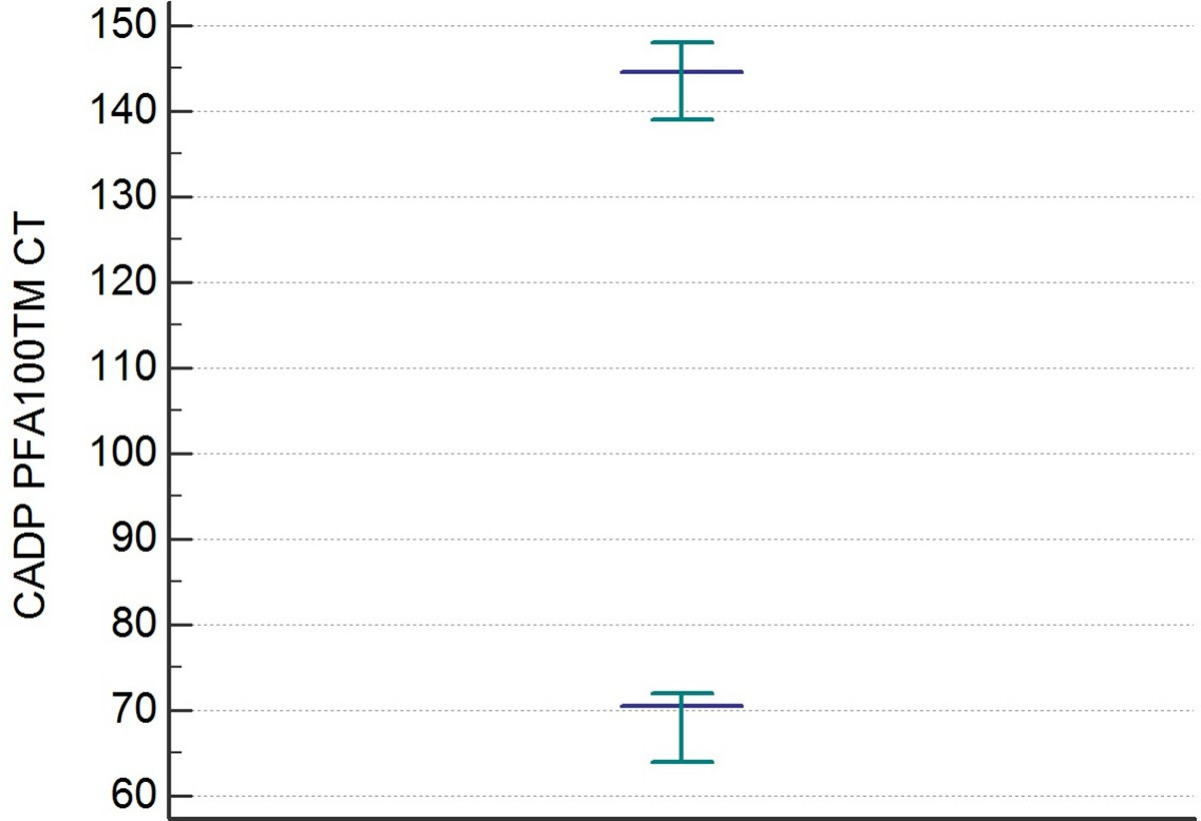
Reference intervals of CADP PFA-100 CT determined by non-parametric percentile method (CLSI C28-A3).

The distributions of the CEPI CT and CADP CT values differed significantly from the Gaussian curve (p< 0,001). After an evaluation of the data by the Dixon test, there were no outliers. The results of the statistical analysis of CEPI CT and CADP CT are shown in Table 2. The RIs of the CEPI CT and CADP CT determined by the 95% Central Interval and 90% CI for upper and lower limit calculated by the two methods (parametric and non-parametric) Table 3. The Figs 1 and 2 illustrate the RIs respectively for CEPI and CADP CT PFA100 determined by the non-parametric method.

**Table 2:** Statistical analysis of CEPI CT and CADP CT PFA-100.

Measurements	CEPI CT	CADP CT
**Sample size**	218	218
**Lowest value**	65.00	63.00
**Highest value**	230.00	150.00
**Arithmetic mean**	142.71	102.64
**Median**	140.00	102.00
**Standard déviation**	31.07	18.84
**Coefficient of Skewness**	0.35 (p = 0,03)	0.40 (p = 0,01)
**Coefficient of Kurtosis**	- 0.38 (p = 0,19)	- 0.25 (p = 0,46)
**D’Agostino-Pearson test**		
• **For normal distribution**	Reject Normality (p = 0.04)	Reject Normality (p = 0.04)
• **After logarithmic transformation**	Accept Normality (p = 0.51)	Accept Normality (p = 0.54)

**Table 3:** Reference intervals of CEPI CT and CADP CT, double-sided.

	CEPI CT (sec)	CADP CT (sec)
**A. Method based on Normal distribution** after logarithmic transformation	
Lower limit	90	70
90% CI	87 to 94 sec	68 to 73
Upper limit	214	144
90% CI	205 to 224	139 to 150
**B. Non-parametric percentile method (CLSI C28-A3)**	
Lower limit	91	70
90% CI	80 to 97	64 to 72
Upper limit	207	144
90% CI	200 to 216	139 to 148

CI: Confidence Interval

### Comparison of the local interval with that of the manufacturer and the foreign populations

As observed in Table 4, similar studies were conducted in Western and Asian populations [6–11]. The RIs provided by the manufacturer of CEPI CT and CADP CT in 3.8% buffered citrate are respectively [84–160] sec and [68–121] sec established on 127 healthy adults. In our comparative study of the RIs, we showed the proportions of our subjects which are classified by the RIs of the manufacturer and other foreign studies as having an abnormality of primary hemostasis in Table 5.

**Table 4:** Reference intervals of PFA-100 closure times from six reports that studied large numbers of healthy individuals.

	Mamman et al.	Ortel et al.	Böck et al.	Haulbelt et al.	Korinkova et al.	Cho et al.	Current Study
**Country**	USA	USA	Germany	Germany	Slovakia	Korea	Algeria
**Number of subjects**	206	176	309	120	50	120	218
**Citrate Concentration**	0.129M (3.8%)	0,129M (3.8%)	0.105M (3.2%)	0.129M (3.8%)	0.105M (3.2%)	0.129M (3.2% & 3.8%)	0.129M (3.8%)
**% of Blood group O**	NC	NC	NC	43.3	46	30	42.7
**Reference Intervals**							
** Method of calculation**	90% CI	NC	90% CI	90% CI	Mean ± SD	95% CI	95% CI
** CT CEPI sec**	94–191	94–193	82–150	93–223	86–199	82–182	91–207
** CT CADP sec**	72–120	71–118	62–100	65–117	42–119	62–109	70–144

NC: No Comment; CI: Central Interval

**Table 5:** Comparison of the local reference values of the CT EPI and CT CADP with those predefined by the manufacturer and other foreign populations.

Parameters Pre-defined		CEPI CT			CADP CT	
By:	Normal	Abnormal	p Value	Normal	Abnormal	p Value
**Manufacturer SIEMENS**	158 (72.5%)	60 (27.5%)	<0.0001	182 (83.5%)	36 (16.5%)	<0.0001
**Mamman et al. USA 1998**	202 (92.7%)	16 (7.3%)	0.023	182 (83.5%)	36 (16.5%)	<0.0001
**Ortel et al. USA 2000**	203 (93.1%)	15 (6.9%)	0.036	179 (82.1%)	39 (17.9%)	<0.0001
**Haulbelt et al. Germany 2005**	217 (99.5)	1 (0.5%)	NS	175 (80.3%)	43 (19.7%)	<0.0001
**Cho et al. Korea 2008**	192 (88.1%)	26 (11.9%)	0.0001	145 (66.5%)	73 (33.5%)	<0.0001

## Discussion

We designed this prospective study according to IFCC–CLSI guidelines for the de novo establishment of RIs of the PFA-100 CT in Algerian adults after standardization of the preanalytical variables. The strength of the present study is the sample size. To our knowledge, this report is the first to determine the RIs of PFA-100 CT in Algeria. Clinical laboratories can validate the local RIs depending on local conditions. The results of our survey show that the local RIs differ from Western and Asian populations. The upper limits for CTs were higher in our study. Using the RIs of the foreign population shows that many healthy Algerian adults could be incorrectly identified as having primary hemostatic defect. Hence, the RIs determined for another population are inappropriate for our population. The ethnic, genetic, dietary and environmental factors influence the pathophysiology of hemostasis and thrombosis [12–14]. Variations in the studies’ methods reported would also lead to different RIs [6–11]. Furthermore, the high prevalence of blood group O among African may also explain the difference RIs between populations [15]. Individuals with blood group O have longer PFA-100 CT [16]. The PFA-100 CT strongly depends on the vWF plasma level, which is lower in blood group O than in other blood groups [17, 18]. Under high shear stress, vWF acts as the mediator for both platelet adhesion and aggregation [19]. Lower levels of vWF affect the formation of a hemostatic plug and typically prolong PFA-100 CT [20]. Our result confirms the influence of ABO blood group on PFA-100 CT. Therefore, it would be more suitable to use these results rather than the RIs from foreign countries to eliminate the variability between local laboratories. It is obvious from this study that there was a need to establish site-specific RIs PFA-100 CT applicable to our population.

## Conclusion

The RIs provided by the kit manufacturers and scientific literature are foreign to the local population. Therefore, the determination of RIs for CT PFA-100 in the local laboratory has an irreplaceable role for further use of PFA-100 in everyday clinical practice. This report is the first to determine the RIs of PFA-100 CT in a well-characterized healthy Algerian population. Thus, other Algerian clinical laboratories could adopt local RIs after verification or validation. These improve the accuracy of diagnosis and patient care in Algeria.

## Supporting information

S1 Table Data collection of 218 healthy individuals. (XLSX)
